# Small bowel neuroendocrine tumors: An analysis of clinical presentation, diagnostic workup and surgical approach—A single center retrospective study

**DOI:** 10.3389/fsurg.2023.1072435

**Published:** 2023-04-03

**Authors:** Veronika Kroepfl, Ruben Bellotti, Elisabeth Gasser, Katharina Esswein, Hannah Esser, Reinhold Kafka-Ritsch, Dietmar Öfner, Alexander Perathoner

**Affiliations:** Department of Visceral, Transplant and Thoracic Surgery, Center of Operative Medicine, Medical University of Innsbruck, Innsbruck, Austria

**Keywords:** neuroendocrine tumors, small-bowel neuroendocrine tumors, diagnostic workup, surgical treatment, oncological outcome

## Abstract

**Background:**

Neurocrine neoplasms (NEN) of the small bowel (SBNEN) are a rare entity and mostly asymptomatic. The aim of this study was to explore trends in the clinical presentation, diagnostic workup, surgical approach and oncological outcome in patients with SBNEN at our surgical department.

**Materials and methods:**

All patients who underwent surgical resection for SBNEN from 2004 to 2020 at our department were enrolled in this single center retrospective study.

**Results:**

A total of 32 patients were included in this study. In most cases, the diagnosis was based on incidental findings during endoscopy or radiographic imaging (*n* = 23; 72%). Twenty cases had a G1 tumor and 12 cases a G2 tumor. The 1-, 3- and 5-year overall survival (OS) were 96%, 86% and 81%, respectively. Patients with a tumor more than 30 mm had a significantly lower OS (*p* = 0.01). For G1 tumors, the estimated disease-free survival (DFS) was 109 months. Again, the DFS was significantly lower when the tumor had more than 30 mm in diameter (*p* = 0.013).

**Conclusion:**

Due to the mostly asymptomatic presentation, the diagnostic workup can be difficult. An aggressive approach and a strict follow-up seem to be important for the oncological outcome.

## Introduction

Neuroendocrine neoplasms (NEN) can arise from neuroendocrine cells throughout the whole body (1), with the majority originating from neuroendocrine cells in the small bowel and pancreas (2).

The WHO classification and grading system for gastroenteropancreatic NEN was updated in 2017 and uses the Ki-67 Index to grade the tumors (3). Well-differentiated NEN are described as NET Grade 1 (G1) with a Ki-67 Index ≤3% and NET G2 with a Ki-67 Index 3%–20% and well-differentiated NET G3 with Ki-67 Index >20%. Poorly differentiated NEN are described as NEC G3 with a Ki-67 Index of more than 20% (3–5). Ki-67 and mitotic index are markers for cell-proliferation (6).

Small-bowel NEN (SBNEN) account for approximately 17% of all neuroendocrine tumors (7). The incidence of SBNEN has recently increased due to improved diagnosis of early stage disease and is now estimated to be 1.05 per 100,000 persons (8).

Clinically, SBNEN can present with the classical trial of flushing, diarrhea and wheezing due to hormone-active tumors especially in the presence of liver metastases (9, 10). These secretions are not metabolized by the liver, remain active and enter the systemic circulation (4). However, most SBNEN do not show any hormone-related symptoms and they are rather slow growing tumors, whose delayed clinical manifestation is mostly tumor-mass-related, e.g., intestinal obstruction or bleeding due to local invasion (10).

In SBNEN the diagnostic strategy strongly depends on the clinical presentation. In the case of a classical carcinoid syndrome, biochemical analysis can aid in confirming the diagnosis. One of the markers indicative of NEN is Chromogranin A (CgA), a protein secreted by cells with autocrine, paracrine or endocrine activity (4, 9). Although it has limited sensitivity and specificity (71% and 50%, respectively), CgA remains the only relevant diagnostic marker (11). Additionally, CgA has a role as tumor surveillance marker after resection (9).

For further work-up, imaging modalities can be used to evaluate anatomical location (CT-scan, ultrasound). Functional analysis using a radio-labeled somatostatin-analog in combination with radiological imaging, such as ^68^Gallium-DOTA-TOC PET CT scans, helps to determine disease extent throughout the whole body (5). Endoscopic strategies can be attempted if the primary tumor cannot be detected. As around 50% of SBNEN occur in the ileum, capsule endoscopy may facilitate the diagnostic work-up (7).

The therapeutic gold standard for SBNEN is a surgical treatment consisting of segmental small-bowel resection or right-sided hemicolectomy (for distal ileal tumors) with resection of regional mesenteric lymph nodes (7, 12). R0-resection should be attempted. To achieve this, no macroscopic nor microscopic tumor remains in the situs. Surgical treatment furthermore includes careful inspection of the peritoneal cavity to exclude potential peritoneal and liver metastasis (1). In the case of oligometastatic liver disease and limited to G1 and G2 differentiation, patients profit from simultaneous or two-stage surgical resection of those and/or local ablative strategies to achieve R0 situation (12–14).

In this study we attempt to evaluate the clinical presentation, the diagnostic workup and the surgical approach of SBNEN at our surgical department over a period of 16 years.

## Materials and methods

### Study design and data collection

All patients who underwent surgical resection for small-bowel tumors at the Department of Visceral, Transplant and Thoracic Surgery Innsbruck from February 2004 until January 2020 were included into a retrospective study. The study protocol was approved by the local ethics committee (Vote number: 1435/2021). Exclusion criteria were defined as a benign disease and/or non-SBNET. After enforcing exclusion criteria, 32 patients were left for statistical analysis.

If the diagnosis was not due to an intestinal obstruction or bleeding leading to an emergency operation, all patients received a PET-CT scan. CgA was analyzed on laboratory testing prior to operation. All patients were discussed in the local interdisciplinary tumor board before and after surgical treatment (if not an emergency operation). If adjuvant therapy was not recommended, all patients received a strict follow-up regimen, which included an appointment every 3 months for the first 2 years. This was extended to a 6-monthly follow-up for further 3 years. Every follow-up included laboratory testing of CgA. Once a year, patients received a PET-CT scan.

At our institution a chemotherapeutic regime is only recommended in case of progression under other classical specific therapies, like somatostatine, PRRT and everolimus.

### Demographic and clinical variables

Data collected from medical records included patient demographic data such as age, sex, Charlson comorbidity index, clinical data such as clinical presentation, imaging and biochemical evaluation results, history of pre- and/or postoperative chemotherapy, duration of hospital stay and surgical approach. Postoperative complications were graded according to the Clavien-Dindo-classification (15) and recorded as minor (grade I–II) or major complications (III–IV).

Tumor variables, such as tumor localization, lymph node and metastasis (TNM) classification, tumor grading, the diameter of the largest lesion, Ki-67 index and recurrence were analyzed. Biologic markers (serum CgA) were recorded.

Overall survival (OS) was calculated as the time from tumor resection to the date of death or last follow-up visit (FU). Disease-free survival (DFS) was defined as the time from initial clearance of all tumor deposits (primary, metastases) to the first recurrence at any site.

### Statistical analysis

A descriptive analysis was performed for all study variables. Absolute and relative frequencies were reported for categorial data, median and range were reported for continuous variables. Differences in categorical variables were investigated by Chi-square- and *t*-test. Kaplan Meier Method was used for survival analysis. Log-rank test was conducted to measure differences in survival variables. Two-tailed *p*-values less than 0.05 were considered statistically significant. IBM SPSS Statistics 26 (IBM Corporation, Armonk, NY, USA) was used for statistical analysis.

## Results

### Study population

The study population consists of 32 patients, 15 patients were female. The study population's median age at date of surgery was 64 years (range 26–87). The median age of female patients was 63 years, whereas the median age of male patients was 65 years. The clinical data are shown in Table 1.

**Table 1:** Demographic and tumor characteristics stratified by gender.

	Total	Female (*n* = 15)	Male (*n* = 17)
Age	64 (26–87)	63	65
**Presentation *n* (%)**			
Ileus	5 (16%)	2 (13%)	3 (18%)
Diarrhea	2 (6%)	1 (7%)	1 (6%)
Flushing syndrome	1 (3%)	1 (7%)	0
Incidental findings	24 (75%)	11 (73%)	13 (76%)
**Localization primum *n* (%)**			
Duodenum	9 (28%)	3 (20%)	6 (35%)
Jejunum	2 (6%)	2 (13%)	0
Ileum	21 (66%)	10 (67%)	11 (65%)
**Grading *n* (%)**			
G1	20 (63%)	5 (33%)	15 (88%)
G2	12 (37%)	10 (67%)	2 (12%)
**pT *n* (%)**			
1	7 (22%)	3 (20%)	4 (24%)
2	9 (28%)	3 (20%)	6 (35%)
3	9 (28%)	4 (27%)	5 (29%)
4	6 (19%)	4 (27%)	2 (12%)
x	1 (3%)	1 (6%)	0
**pN *n* (%)**			
0	9 (28%)	2 (13%)	7 (41%)
1	21 (66%)	12 (80%)	9 (53%)
x	3 (6%)	1 (7%)	1 (6%)
**pM *n* (%)**			
0	22 (69%)	7 (47%)	15 (88%)
1	10 (31%)	8 (53%)	2 (12%)
**Tumor diameter *n* (%)**			
≤30mm	23 (72%)	9 (60%)	14 (82%)
>30mm	9 (28%)	6 (40%)	3 (18%)
**Recurrence *n* (%)**			
Yes	12 (38%)	6 (40%)	6 (35%)
No	20 (62%)	9 (60%)	11 (65%)

**Table 2:** Survival analysis for factors affecting patients' OS using a COX proportional hazard model.

Factor	Univariable		Multivariable		
	Median survival (months)	*p*	*p*	Hazard ratio	95% CI
Emergency surgery	40.0	0.010	0.015	23.196	1.839–292.589
Dimension >30 mm	82.0	0.010	0.012	18.874	1.888–188.704

**Table 3:** Survival analysis for factors affecting patients' DSF using a Cox proportional hazard model.

Factor	Univariable		Multivariable		
	Median survival (months)	*p*	*p*	Hazard ratio	95% CI
Emergency surgery	152.0	0.895	0.510	2.096	0.232–18.928
Dimension >30 mm	35.0	0.013	0.020	5.027	1.291–19.570

Localization of the primary included ileum (*n* = 21; 66%), duodenum (*n* = 9; 28%) and jejunum (*n* = 2; 6%). The diagnosis was based on incidental findings in 23 cases (72%) during an endoscopic procedure or radiographic imaging. In the remaining patients, diagnostic evaluation was initiated upon clinical symptoms such as intestinal obstruction (*n* = 5; 16%), diarrhea (*n* = 2; 6%), B-symptoms (*n* = 1; 3%) and flushing syndrome (*n* = 1; 3%). In four cases (13%), an emergency operation had to be performed.

As per histopathological grading tumors were classified as G1 in 20 cases (63%) and G2 in 12 cases. There was no G3 tumor. Male patients displayed a higher percentage (88%) of G1 tumors when compared to female patients, whereas female patients displayed a higher rate (67%) of G2 tumors.

Most patients had a T2 or T3 tumor (*n* = 9; 28%). In 23 patients, primary tumor lesions were smaller than 3 cm (72%) and distant metastases were diagnosed in 10 cases (31%). Considering tumor dimension larger than 3 cm, we could observe a significant correlation with local lymphatic-vessel invasion (*p* = 0.029) and perineural invasion (*p* = 0.08), but not with local vascular invasion (*p* = 0.409).

According to the guidelines, CgA was analyzed on laboratory testing before the surgical procedure (*n* = 21) and was elevated in 38% (*n* = 12). Our department's normal CgA levels range from 0.0 to 108 µg/L. This was only not performed in exceptional cases. For example, in emergency surgeries due to intestinal obstruction or CgA was not available for statistical analysis (missing *n* = 11).

The surgical approach was open in 30 cases. Laparoscopic resection was performed on only two patients. The majority of the patients received an oncological right-sided hemicolectomy (*n* = 13; 40%) or a segmental small bowel resection (*n* = 12; 38%) followed by tumor extirpation (*n* = 5; 16%), a pylorus-preserving pancreaticoduodenectomy (PPPD) and distal gastrectomy (*n* = 1; 3%) due to a tumor in the proximal duodenum. R0-resection was achieved in all but five patients (*n* = 27, 84%).

One patient received a platin-based neoadjuvant chemotherapy actually as a treatment for a concurrent histologically verified bronchial adenocarcinoma. Six patients received postoperative therapy: this included in three cases somatostatines and in three cases PRRT.

Post-operative complications occurred in 10 patients. Six cases were graded as mild and four as severe complications according to the Clavien–Dindo Classification. Severe complications included anastomotic leakage, postoperative hemorrhage, pleural effusion and postoperative cholecystitis with a consecutive operation (all *n* = 1).

### Survival analysis and oncological outcome

**Figure 1:**
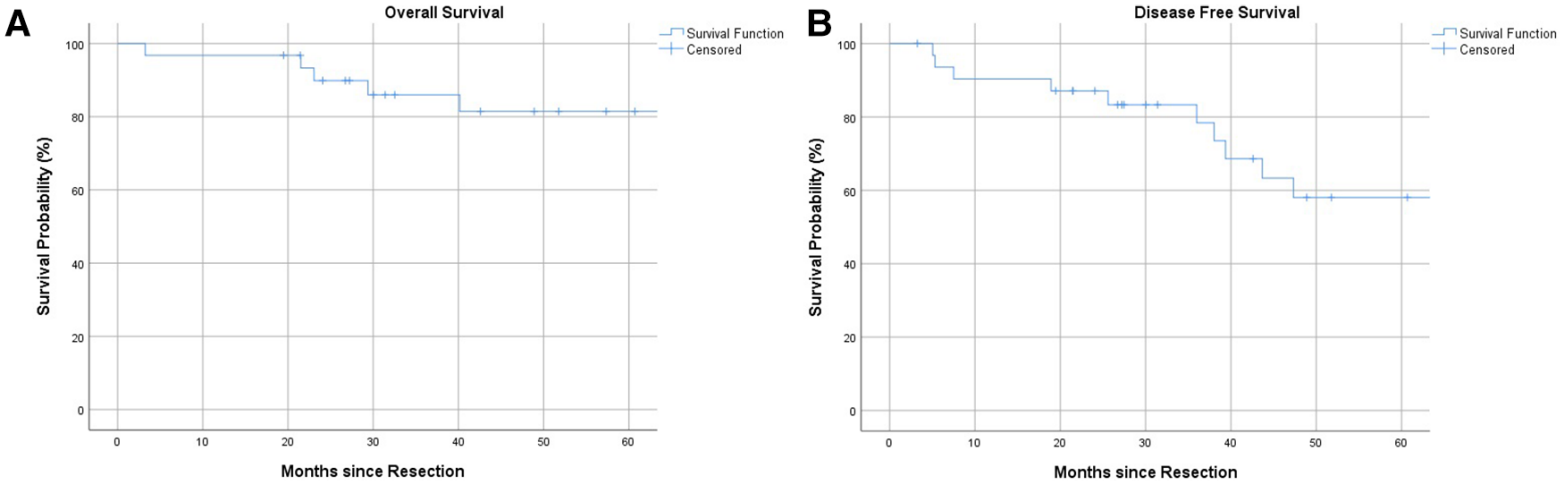
(**A**) 1-, 3- and 5-year overall survival. (**B**) 1-, 3- and 5 year disease-free survival.

**Figure 2:**
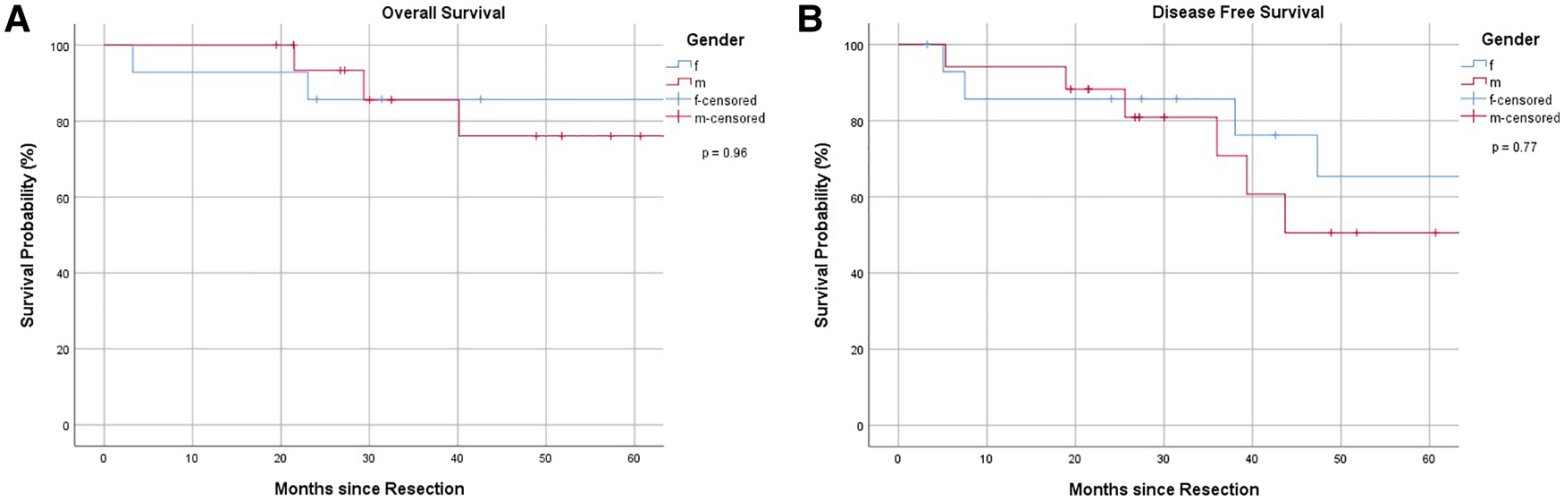
(**A**) Overall survival, stratified by gender. (**B**) Disease-free survival, stratified by gender.

Overall 1-, 3- and 5-year survival were 96%, 86% and 81%, respectively (Figure 1A). Estimated survival for female patients was 136 vs. 142 months for male patients and did not show statistical significance (Figure 2A). We found no differences with respect to tumor grading (*p* = 0.47), nodal status (*p* = 0.72) and synchronous metastases (*p* = 0.87) regarding survival. Compared to the tumor size, the log-rank test revealed a significantly lower patient survival for patients with a tumor >30 mm (156 vs. 82 months; *p* = 0.01). Equally, patients undergoing an emergency surgery showed shorter OS (40 vs. 162 months; *p* = 0.01) as seen in Table 2.

The 1-, 3- and 5-year DFS were 90%, 78% and 58%, respectively with no statistical difference in female and male patients (*p* = 0.77) as Figures 1B, 2B show. The recurrence rate was 35.5% within the follow-up period. The estimated DFS for patients with G1 tumors was 109 months. For G2 tumors it was 94 months (*p* = 0.55). Again, when compared to the tumor size, patients with a tumor >30 mm had a significantly worse DFS (123 vs. 35 months; *p* = 0.013). We could not find a significant difference in nodal status (*p* = 0.21), synchronous metastases (*p* = 0.1) and performing emergency surgery (*p* = 0.895) regarding the DFS.

Local recurrence was observed in one out of 12 cases (8%), whereas the most frequent type of distant metastases were liver metastases (*n* = 6; 50%), followed by metastases in the mesentery (*n* = 2), pulmonic, ovarian and multiple metastases (all *n* = 1). In seven of these cases, CgA levels were elevated on laboratory testing. In 10 of these patients, mesenteric lymph node metastases were diagnosed at the time of resection.

## Discussion

This retrospective study evaluated clinical presentation, surgical treatment, short-term surgical outcome and long-term oncological outcome in a cohort of patients who underwent surgical resection of SBNET with homogenous grading. In our study, the majority of diagnoses (*n* = 24, 75%) were based on incidental findings obtained, e.g., during a medical check-up. Presentations due to symptoms caused by hormone secretion were relatively uncommon. These findings are in accordance with other available data evaluation (16–18). Hormone-secretion related symptoms have been described by Terminassian et al. (12% diarrhea, 7% flushing syndrome), which supports the findings of this study (18). The histopathological evaluation found the majority of the tumor lesions to be ≤3 cm in diameter, which correlates with the literature (≤3 cm in 75.9% vs. >3 cm in 24.1%) (19).

Mesenteric lymph node metastases were detected in 21 patients (65.6%), of which ten presented with tumor recurrence. Landry et al. and Pasquer et al. demonstrated that resection of mesenteric lymph nodes improves survival by lowering disease recurrence, preventing complications like obstruction and ischemia and improving the survival rate (17, 20). This highlights the importance of a radical surgical regime with dissection of the mesenteric lymph nodes to achieve a better oncological outcome.

In our study, we observed only a small number of patients with an early tumor stage (four patients in UICC I and four patients in UICC II). Yet, despite the low percentage of early tumor stages and the higher percentages of stages III and IV in our cohort, we did not observe a higher recurrence rate, which seems to be similar to the available literature (35.5% in our cohort vs. 31% in the literature) (21). In contrast to the available literature, in our cohort, tumor grading similar to tumor stage had no significant impact on recurrence rate, where the risk of recurrence is described to increase with the stage of disease (18, 21). This can potentially be explained by the high number of well-differentiated G1 NET (*n* = 20, 62.5%) and small sample size of our study. G2 grade was observed in 12 patients and there was no G3 tumor or neuroendocrine carcinoma. In the literature, the percentage of of G1 tumors ranges from 61% to 96.6% (17, 18).

We did not detect gender-based differences in OS or DFS accordingly to current literature (18).

Distant metastases are described to have a low prognostic impact on the DF (21). In our cohort, the analysis of DFS did not show any significant difference with or without synchronous metastases (*p* = 0.1) but can be explained by the small sample size. The 5-year OS was 58%, indicating to select patients who can take advantage of a radical surgical approach. A radical surgical approach should be attempted to gain R0 resection and avoid tumor recurrence. This should include a complete oncological resection of the primary with resection of all regional lymph nodes (17, 21, 22). The timing of surgery and surgical approach depends on clinical presentation. As seen in our cohort, a radical surgical approach is crucial for the oncological outcome, with only 38% resulting in tumor recurrence. In our cohort, most patients received a radical oncological resection, which included a hemicolectomy or segmental small-bowel resection. Concerning synchronous liver metastases, two patients were radically resected with either hemihepatectomy or atypical liver resection. In two cases radical local ablation with stereotactic radiofrequency (RFA) was performed. Only one patient with multiple liver lesion was treated in a palliative setting with platin-base chemotherapy.

In our cohort, a tumor diameter with more than 3cm is a negative prognostic factor as seen in Tables 2, 3. This could be explained due to higher frequencies of perineural invasion as well as local lymph node invasion in these cases. Curiously, not the nodal status not the presence of synchronous metastases and even the tumor grading showed any correlation with the patients’ prognosis. Of note, these results are not simply explained by the small sample size. It should be also considered that our series consists of only well-differentiated SBNET (G1 and G2) which show good response to multimodal strategies contemplating local and systemic treatment as well. Not only a multimodal strategy but also a multidisciplinary approach was shown to be clinically relevant in the management of NEN patients (19).

Tumor recurrence was detected during oncological follow-up, on the one hand due to elevated CgA-levels, on the other hand, *via* imaging (PET-CT, CT-scan). Treatment of tumor-recurrence was with either a radical surgical approach, RFA or systemic chemotherapy. Only one patient did not receive specific therapy for tumor recurrence due to advanced age and comorbidities. As Watzka et al. described in their work, patients benefit from resection of hepatic metastases with respect to survival rate (88.5% 5-year survival rate with R0/R1 resection vs. 69.1% with R2- or no resection of hepatic metastases) (19). Elevated CgA levels are reported to associate with shorter survival (17, 18, 23). As seen in our cohort, laboratory testing of CgA-levels during oncological follow-up is an excellent tool to early diagnose tumor recurrence, as elevated levels correlate with tumor burden (24). The measurement of CgA is cheap and straightforward to achieve and can be performed at the general practitioner's office.

Especially because SBNEN represent a rare entity with rising incidence (7, 8, 17), it is crucial to address the primary symptoms at diagnosis, the therapeutic management and the long-term oncological outcome of this special entity. Due to their relative seldom incidence, previous literature data are quite inhomogeneous (Table 4) and nowadays further studies, in particular randomized controlled trials, are needed to better uniform the clinical management of these entities (25–34).

**Table 4:** SBNEN series described in literature.

Author (year)	Number of patients	Median OS (months)	5-year survival (%)	Median DFS
Søreide et al. (1992) (25)	53	139	n.a.	n.a.
Schindl et al. (2002) (26)	58	n.a.	76.0	n.a.
Givi et al. (2006) (27)	66	108	81	54
Strøsberg et al. (2009) (28)	100	110	n.a.	n.a.
Ahmed et al. (2009) (29)	209	119	74	n.a.
Norlén et al. (2012) (30)	493	n.a.	75	n.a.
Randle et al. (2013) (31)	1,360	143	73.9	n.a.
Mosquera et al. (2016) (32)	53	n.a.	81.1	n.a.
Noujaim et al. (2017) (33)	16	53.5	n.a.	n.a.
Zaidi et al. (2019) (34)	199	39	n.a.	n.a.

This single-center retrospective analysis certainly has its limitations. Due to the small sample size, we could not describe significant differences in survival between various parameters in detail. As described above, tumor size was one of the most critical factors regarding the OS and DFS. Furthermore, for us an aggressive surgical approach seems crucial for patients’ disease-free and overall survival.

In addition to those findings, a strict and consequent follow-up, including laboratory testing of CgA before primary surgery and during each follow-up appointment and repeated imaging with at least a PET-CT scan once a year, helps to detect tumor recurrence early.

## Conclusion

SBNEN are mostly asymptomatic; therefore, preoperative diagnostic work-up is complex. Diagnosis often happens by incident. According to our results, an aggressive surgical regimen and a strict follow-up period seem to be valuable therapeutic strategies to achieve a favorable oncological outcome.

## Data Availability

The raw data supporting the conclusions of this article will be made available by the authors, without undue reservation.
